# Synthesis and Biological Evaluation of Thieno[3,2-*d*]- pyrimidinones, Thieno[3,2-*d*]pyrimidines and Quinazolinones: Conformationally Restricted 17β-Hydroxysteroid Dehydrogenase Type 2 (17β-HSD2) Inhibitors

**DOI:** 10.3390/molecules18044487

**Published:** 2013-04-16

**Authors:** Enrico Perspicace, Sandrine Marchais-Oberwinkler, Rolf W. Hartmann

**Affiliations:** 1 Pharmaceutical and Medicinal Chemistry, Saarland University, Campus C23, D-66123 Saarbrücken, Germany; E-Mails: e.perspicace@mx.uni-saarland.de (E.P.); s.marchais@mx.uni-saarland.de (S.M.-O.); 2 Helmholtz Institute for Pharmaceutical Research Saarland (HIPS), Campus C23, D-66123 Saarbrücken, Germany

**Keywords:** thieno[3,2-*d*]pyrimidinones, thieno[3,2-*d*]pyrimidines, quinazolinones, conformationally restricted analogues, enzyme inhibitors, 17β-hydroxysteroid dehydrogenase type 2, osteoporosis

## Abstract

In this study, a series of conformationally restricted thieno[3,2-*d*]pyrimidinones, thieno[3,2-*d*]pyrimidines and quinazolinones was designed and synthesized with the goal of improving the biological activity as 17β-hydroxysteroid dehydrogenase type 2 inhibitors of the corresponding amidothiophene derivatives. Two moderately active compounds were discovered and this allowed the identification of the biologically active open conformer as well as the extension of the enzyme binding site characterisation.

## 1. Introduction

Osteoporosis is a systemic skeletal disease characterised by reduction of bone mineral density and deterioration of the bone microarchitecture leading to an increase in bone fragility and thus elevated bone fracture risk [1]. The decrease in active steroid levels like estradiol (E2) and testosterone (T) is often associated with the development of osteoporosis (e.g., in women at menopause [2]). As this disease affects elderly men and especially women and the population is getting older, it has become a serious public health issue and new therapeutic alternatives have to be developed.

17β-Hydroxysteroid dehydrogenase type 2 (17β-HSD2, (EC1.1.1.51) [3]) belongs to the short-chain steroid dehydrogenase/reductase protein family (SDR). This enzyme catalyses the NAD^+^-dependent oxidation of the potent estradiol (E2) into estrone (E1) as well as testosterone (T) into androstenedione (Δ4-dione). 17β-HSD2 is a membrane-associated protein located in the endoplasmic reticulum and contains 387 amino acids. This enzyme is highly expressed in a wide variety of tissues such as placenta, liver, small intestine, endometrium, urinary tract and to a lesser extend also in kidney, pancreas, colon, uterus, breast, prostate and bone. The 3D-structure of this enzyme remains unknown.

**Figure 1:**
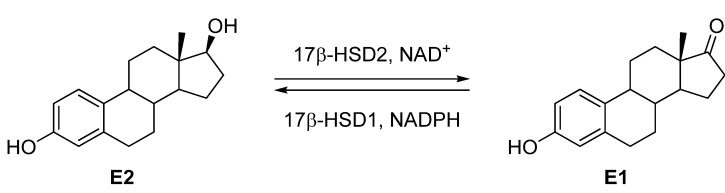
Interconversion of Estradiol (E2) to Estrone (E1) by 17β-HSD2 and 17β-HSD1.

Few 17β-HSD2 inhibitors have been described in the literature [4,5]. The concept of improving bone quality by inhibiting 17β-HSD2 has been validated by Bagi *et al.* in a monkey osteoporosis model [2] using a *cis*-pyrrolidinone derivative as 17β-HSD2 inhibitor [6,7,8]. However, the treatment with this compound led to few effects on bones. Therefore, it is necessary to develop new potent 17β-HSD2 inhibitors with better *in vivo* efficacy, which are selective toward 17β-HSD1, the isoenzyme catalysing the reverse reaction, the reduction of E1 into E2 (Figure 1).

Our group has been working for 20 years on the development of inhibitors of steroidogenic enzymes (e.g., 5α-reductase [9], aromatase [10,11], CYP17 [12,13], CYP11B2 [14,15,16,17], CYP11B1 [18,19]) and in the last years a lot of experience was gained in the design of potent and selective inhibitors of 17β-HSD1 [20,21,22,23,24,25], and 17β-HSD2 [26,27,28,29,30,31,32]. Recently, we described [27] *N*-(3-methoxybenzyl)-5-(3-methoxyphenyl)-*N*-methylthiophene-2-carboxamide (compound **A**, Table 1) and *N*-(3-hydroxybenzyl)-5-(3-hydroxyphenyl)-*N*-methylthiophene-2-carboxamide (compound **B**, Table 1) as 17β-HSD2 inhibitors (63% and 70% 17β-HSD2 inhibition at a concentration of 1 µM, for **A** and **B**, respectively). Both compounds show a good selectivity toward 17β-HSD1 (0% and 21% inhibition at 1 µM, respectively). Compounds **A** and **B** consist of a central thiophene moiety connected to a substituted phenyl ring on one side and to a substituted benzyl on the other side linked to the thiophene via a methylated carboxamide bond. **A** and **B** show some flexibility at this amide bond, allowing for several conformations.

In the ^1^H-NMR spectra of **A** or **B** it can be seen that the peaks corresponding to N-Me group (3.15/3.16 ppm) and to the CH_2_ of the benzyl group (4.76/4.79 ppm) appear as very broad singlets, indicating that in solution at room temperature **A** and **B** exist as a mixture of several conformers in equilibrium. However, when the compounds bind to the enzyme binding site, only one conformer is expected to be present and therefore only one conformer is biologically active.

**Table 1:** *In vitro* 17β-HSD2 and 17β-HSD1 inhibitory potencies of thiophene-2-carboxamide derivatives **A** and **B**. 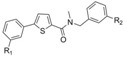

Cmpd	R_1_	R_2_	Percentage of inhibition at 1µM ^a^	
			17β-HSD2 ^b^	17β-HSD1 ^c^
**A**	OMe	OMe	63%	0%
**B**	OH	OH	70%	21%

^a^ Mean value of three determinations, standard deviation less than 15%. ^b^ Human placental, microsomal fraction, substrate E2 [500 nM], cofactor NAD+ [1500 nM]. c Human placental, cytosolic fraction, substrate E1 [500 nM], cofactor NADH [500 nM].

Rigidification is an often applied method to increase the potency of active molecules. In addition to freezing a compound in one conformation when several exist, it is also a good strategy to identify the active conformation of a compound when it binds in the active site of a protein. Rigidification can be a useful tool for structural optimisation and also to characterize the binding site in the protein.

The goal of this study was the structural optimization of the 17β-HSD2 inhibitors **A** and **B**, using the rigidification strategy as well as the identification of the active conformation taken by **A** and **B** in the protein. In this study the design and the synthesis of new rigidified 17β-HSD2 inhibitors, mimicking the different geometries which can be adopted by the conformers derived from **A** and **B** as well as the biological evaluation of the synthesized compounds (17β-HSD2 potency as well as selectivity toward 17β-HSD1) are reported.

## 2. Results and Discussion

**Figure 2:**
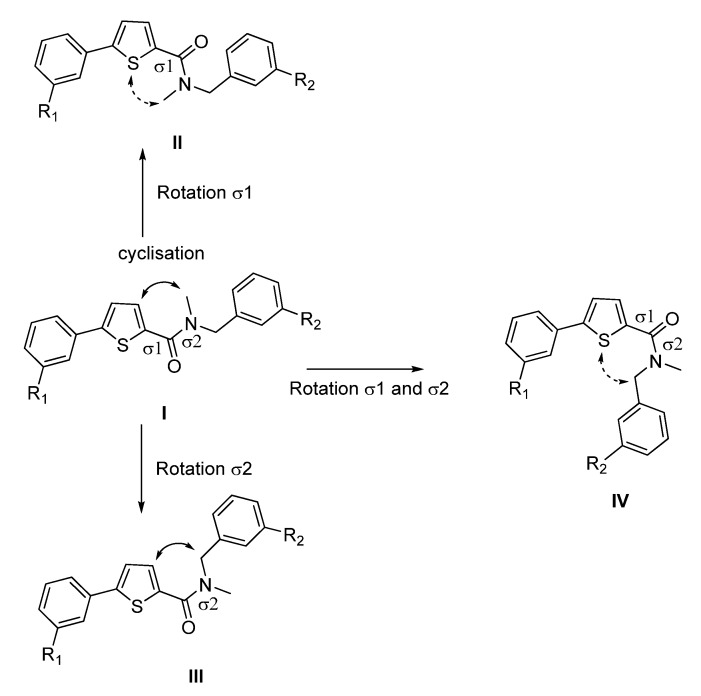
Four major conformers of thiophene-2-carboxamide derivatives **A** and **B**.

**Figure 3:**
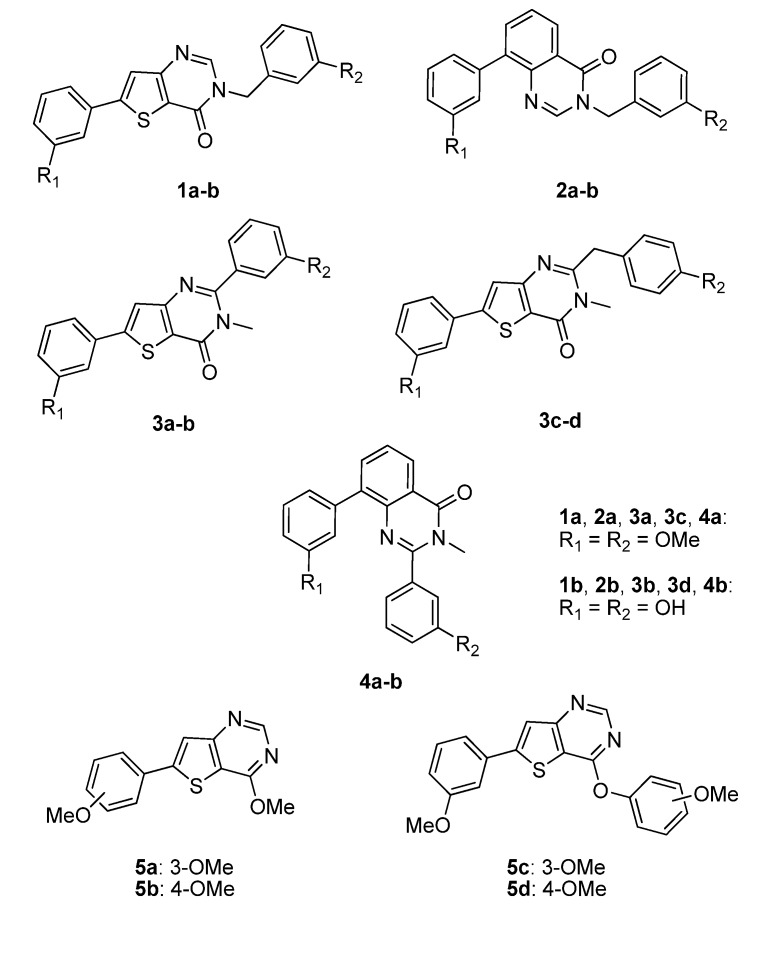
Thieno[3,2-*d*]pyrimidin-4-ones **1a**–**b**, **3a**–**d** and thieno[3,2-*d*]pyrimidine **5a**–**d** as conformationally restrained analogues of conformer **I** and **III**, and quinazolin-4-one **2a**–**b**, **4a**–**b** as conformationally restrained analogues of conformer **II** and **IV**, respectively.

Compounds **A** and **B** show the highest flexibility at the amide bond, involving two sigma bonds (σ1 between the thiophene and the carbonyl moiety and σ2 between the carbonyl and the amino group, Figure 2). Since σ bonds are freely rotatable, rotation about the two sigma bonds (σ1 and σ2) of the amide group leads to four different conformers **I**-**IV** as depicted in Figure 2. The conformational freezing of **A** and **B** can be achieved by cyclisation leading to thiophene- or phenyl-fused derivatives: the thieno[3,2-*d*]pyrimidin-4-one analogues **1a**–**b** and **3a**–**d**, locked conformers **I** and **III**, respectively and quinazolin-4-one derivatives **2a**–**b** and **4a**–**b**,locked conformers **II** and **IV**, respectively (Figure 3). As restrained conformers **II** and **IV** are not possible with the thiophene core (S is bivalent), the heterocycle was replaced by the bioisosteric phenyl ring (Figure 3). In order to investigate the necessity of the benzyl group and its position, compounds **5a**–**d** were designed (conformer **I** or **III**), and synthesized (Figure 3).

**Scheme 1:**
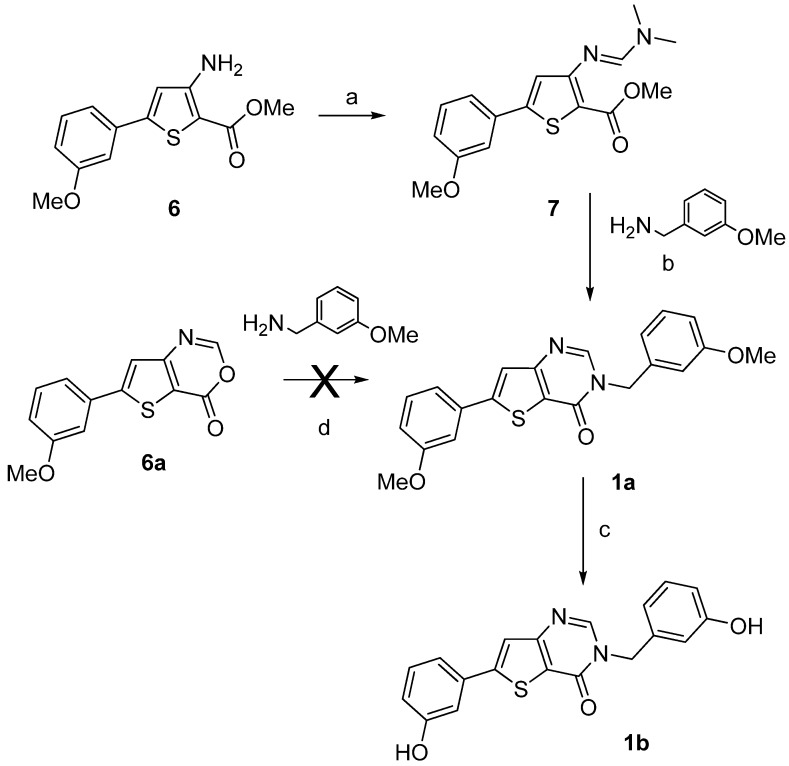
Synthesis of the thieno[3,2-*d*]pyrimidin-4-ones derivatives **1a**–**b**.

Compounds **1a**–**b** were synthesized by a two- or a three-step procedure, respectively, as depicted on Scheme 1, starting from compound **6**. The starting materials methyl 3-amino-5-(3-methoxyphenyl)- thiophene-2-carboxylate (**6**) and 3-amino-5-(3-methoxyphenyl)thiophene-2-carboxamide (**11a**) were synthesized using the multi-step procedure well described in the literature [33]. The condensation of molecule **6** with *N*,*N*-dimethylformamide dimethyl acetal (DMF-DMA) gave methyl 3-dimethyl-aminomethylidene-amino-5-(3-methoxyphenyl)thiophene-2-carboxylate (**7**) in very good yield [34] which was used directly in the next step without purification to afford compound **1a**. The condensation of 6-(3-methoxyphenyl)-4H-thieno[3,2-*d*][1,3]oxazin-4-one (**6a**) with 3-methoxybenzyl amine as an alternative route to produce **1a** failed. After O-demethylation using boron trifluoride methyl sulfide complex BF3.SMe2, the thieno[3,2-*d*]pyrimidin-4(3H)-one **1b** was obtained (Scheme 1).

*Reagents and conditions*: (**a**) DMF-DMA, EtOH, microwave irradiation (100 °C, 80 W, 15 min), 98%; (**b**) DMF, microwave irradiation (100 °C, 80 W, 15 min), 31%; (**c**) BF_3_.SMe_2_, CH_2_Cl_2_, rt, 82%; (**d**) MeOH, reflux.

**Scheme 2:**
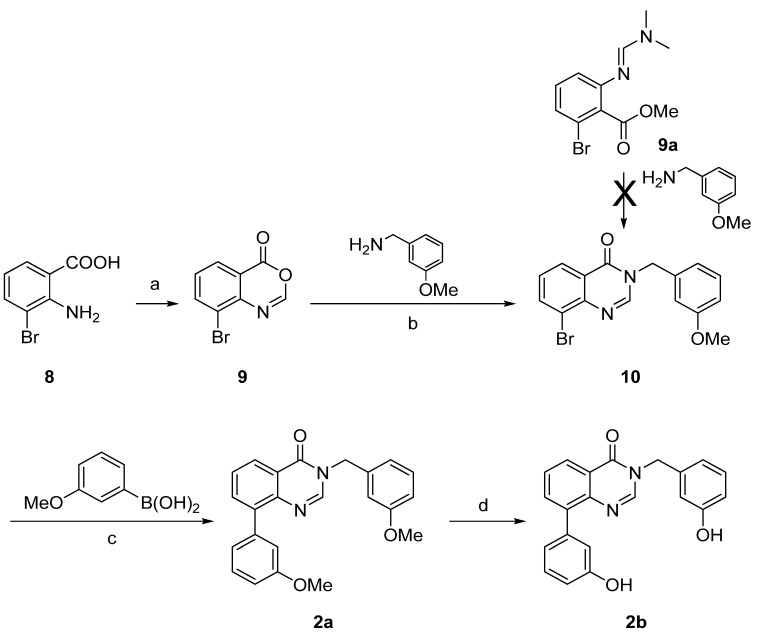
Synthesis of the quinazolin-4-ones derivatives **2a**–**b**.

Synthesis of derivatives **2a**–**b** was achieved by a three- or a four-step reaction pathway starting from the 2-amino-3-bromobenzoic acid (**8**) as shown on Scheme 2. The starting material **8** was prepared as described in the literature [35]. It reacted with triethyl orthoformate [CH(OEt)_3_] to give the cyclized benzoxazin-4-one **9**, which was condensed with 3-methoxybenzylamine to afford the quinazolin-4-one **10** (Scheme 2) [36]. Suzuki-Miyaura cross coupling led to the methoxy derivative **2a** in very good yield. Ether cleavage using the above described procedure afforded the hydroxylated quinazolin-4-one **2b**. It is worth mentioning, that the condensation of methyl 2-bromo-6-{[(1*E*)-(dimethylamino)methylidene]amino}benzoate (**9a**) with the 3-methoxybenzyl amine, which was successfully applied in the synthesis of the thiophene fused **1a**, failed to react to the quinazolin-4-one **10**.

*Reagents and conditions*: (**a**) CH(OEt)_3_, microwave irradiation (160 °C, 80 W, 10 min), 61%; (**b**) dry toluene, reflux, 66%; (**c**) Pd(PPh_3_)_4_, Cs_2_CO_3_, DME/EtOH/H_2_O (1:1:1), microwave irradiation (150 °C, 150 W, 15 min), 98%; (**d**) BF_3_.SMe_2_, CH_2_Cl_2_, rt, 97%.

**Scheme 3:**
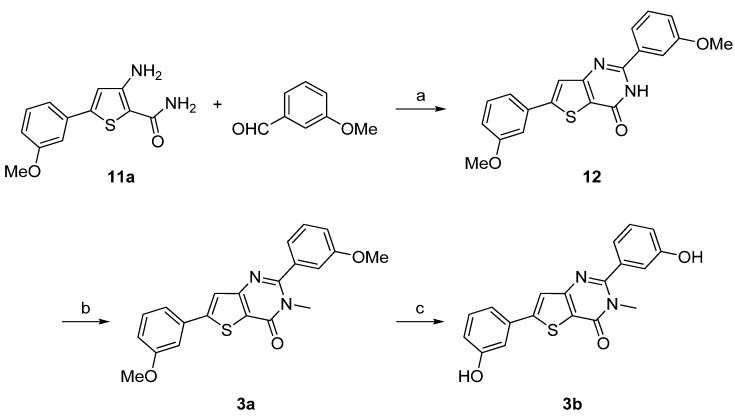
Synthesis of the thieno[3,2-*d*]pyrimidin-4-ones derivatives **3a**–**b**.

**Scheme 4:**
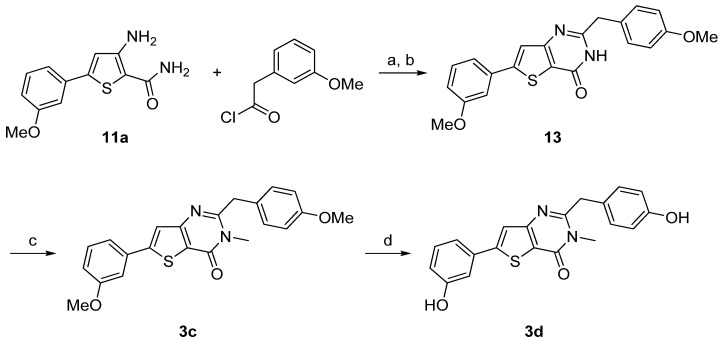
Synthesis of the thieno[3,2-*d*]pyrimidin-4-ones derivatives **3c**–**d**.

Synthesis of **3a**–**d** was performed starting from the 3-amino-5-arylthiophene (**11a**) as depicted in Scheme 3 and Scheme 4 following a two- or a three-step procedure. Compound **11a** was condensed with 3-methoxybenzaldehyde in presence of concentrated hydrochloric acid to give first the hydrogenated thienopyrimidinone which was then autoxidized to the corresponding thienopyrimidinone **12** on heating in acidic medium [37]. Then, *N*-methylation was performed as previously described to afford **3a**. Ether cleavage led to the thieno[3,2-*d*]pyrimidin-4(3H)-one 3b (Scheme 3).

3-Amino-5-arylthiophene (**11a**) was also condensed with 3-methoxyphenylacetyl chloride in presence of triethylamine. The cyclization step was realized with sodium hydroxide to obtain **13**. *N*-methylation was performed using methyliodide in presence of potassium carbonate as base to give **3c** and ether cleavage was accomplished using the same procedure as described above providing **3d** in very good yield (Scheme 4).

*Reagents and conditions*: (**a**) HCl 37%, MeOH, reflux, 38%; (**b**) MeI, K_2_CO_3_, CH_3_CN, reflux, 48%; (**c**) BF_3_.SMe_2_, CH_2_Cl_2_, rt, 57%.

*Reagents and conditions*: (**a**) Et_3_N, dry THF, rt; (**b**) NaOH 2N, few drops of DMF, reflux, 49% (over two steps); (**c**) MeI, K_2_CO_3_, CH_3_CN, reflux, 74%; (**d**) BF_3_.SMe_2_, CH_2_Cl_2_, rt, 98%.

**Scheme 5:**
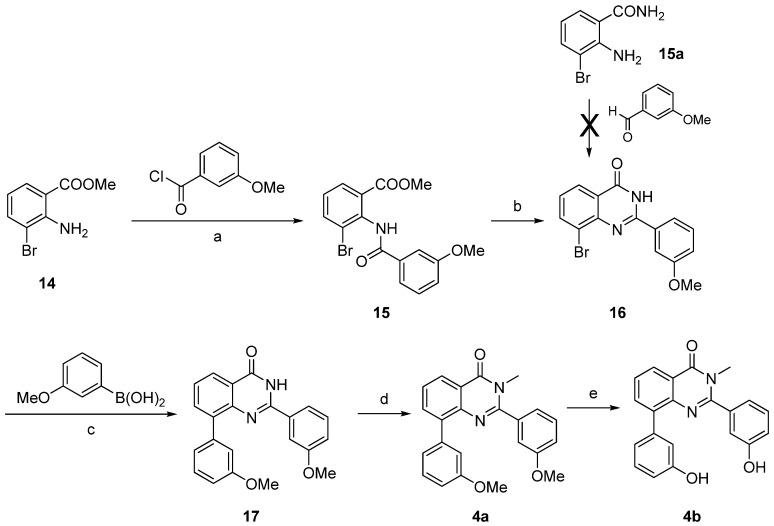
Synthesis of the quinazolin-4-ones derivatives **4a**–**b**.

The 2-substituted quinazolin-4-one **4b** was synthesized starting from 2-amino-3-bromobenzoic acid methyl ester (**14**) (Scheme 5), which was reacted with 3-methoxybenzoyl chloride to afford the amide **15**. This intermediate was condensed with formamide to afford the 2-substituted quinazolin-4-one **16** [38]. Compound **16** could not be obtained from condensation of 2-amino-6-bromobenzamide (**15a**) with 3-methoxybenzaldehyde as described for the synthesis of the thiophene fused derivative **13**, even using different catalysts (*p*-toluenesulfonic acid or sodium metabisulfite). This indicates a different reactivity of the amino group depending on its location on a thiophene or a phenyl ring. Subsequently, the Suzuki-Miyaura cross coupling led to compound **17**, which was then *N*-methylated at the quinazolin-4-one into **4a**. Treatment with BF_3_.SMe_2_ was used to cleave the methoxy groups affording compound **4b**.

*Reagents and conditions*: (**a**) Et_3_N, dry CH_2_Cl_2_, rt, 15%; (**b**) H_2_NCHO, 180 °C, 81%; (**c**) Pd(PPh_3_)_4_, Cs_2_CO_3_, DME/EtOH/H_2_O (1:1:1), microwave irradiation (150 °C, 150 W, 15 min), 77%; (**d**) MeI, K_2_CO_3_, CH_3_CN, reflux, 64%; (**e**) BF_3_.SMe_2_, CH_2_Cl_2_, rt, 98%.

**Scheme 6:**
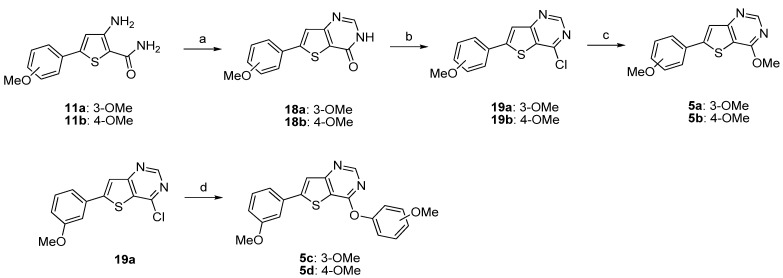
Synthesis of the thieno[3,2-*d*]pyrimidin-4-ones derivatives **5a**–**d**.

The synthesis of fused-compounds **5a**–**b** and **5c**–**d** was performed following two different synthetic routes depicted in Scheme 6. The 3-amino-5-arylthiophene amides **11a**,**b** were condensed with formic acid under microwave irradiation to afford the corresponding thieno[3,2-*d*]pyrimidin-4-one **18a**,**b** as described in the literature [39]. The conversion of these analogues **18a**,**b** to thieno[3,2-*d*]pyrimidines **19a**,**b** was performed using phosphorus oxychloride. The nucleophilic aromatic substitution of **19a**, **19b** was accomplished with sodium methylate and gave the desired 4-methoxy-thieno[3,2-*d*]pyrimidines **5a** and **5b** (Scheme 6). Compound **19a** was reacted with the corresponding sodium methoxyphenolates providing the substituted 4-phenoxythieno[3,2-*d*]pyrimidines **5c**,**d**, respectively (Scheme 6).

The synthesized compounds were tested for their ability to inhibit 17β-HSD2 and 17β-HSD1 using placental enzymes as previously described (compound concentration: 1 µM) [40]. Results are reported in Table 2 as percentage of inhibition. Compounds showing an inhibitory activity below 10% were considered as inactive.

*Reagents and conditions*: (**a**) HCOOH, H_2_SO_4_, 50 °C overnight, **18a**: 76%, **18b**: 98%; (**b**) POCl_3_, microwave irradiation (95 °C, 80 W, 20 min), **19a**: 98%, **19b**: 99%; (**c**) MeONa in MeOH, DMF, rt, 2 h, **5a**: 91%, **5b**: 40%; (**d**) sodium 3-methoxyphenolate or sodium 4-methoxyphenolate, DMF dry, rt, 2 h, **5c**: 88%, **5d**: 87%.

**Table 2:** *In vitro* inhibitory potencies toward 17β-HSD2 and 17β-HSD1 of compounds **1a**–**b**, **2a**–**b**, **3a**–**d**, **4a**–**b** and **5a**–**d**, **12** and **13**.

Compound	Percentage of inhibition at 1 µM ^a^	
	17β-HSD2 ^b^	17β-HSD1 ^c^
**1a**	n.i.	n.i.
**1b**	n.i.	n.i.
**2a**	n.i.	n.i.
**2b**	11%	n.i.
**3a**	n.i.	n.i.
**3b**	36%	50%
**3c**	n.i.	n.i.
**3d**	25%	48%
**4a**	n.i.	n.i.
**4b**	n.i.	n.i.
**5a**	n.i.	n.i.
**5b**	n.i.	n.i.
**5c**	n.i.	n.i.
**5d**	n.i.	n.i.
**13**	n.i.	n.i.
**12**	n.i.	n.i.

^a^ Mean value of three determinations, standard deviation less than 15%. ^b^ Human placental, microsomal fraction, substrate E2 [500 nM], cofactor NAD^+^ [1500 nM]. ^c^ Human placental, cytosolic fraction, substrate E1 [500 nM], cofactor NADH [500 nM]. ^d^ n.i.: no inhibition or inhibition below 10%.

The most active compounds **3b** and **3d** show moderate 17β-HSD2 inhibition (36% and 25%, respectively, Table 2). These two compounds are fused thiophenes and bear two hydroxyphenyl moieties. The corresponding methoxy derivatives are inactive. Compounds **3b**/**3d** both result from the freezing of conformer **III** and differ only in the presence of a phenyl or a benzyl group in position 2 of the pyrimidinone. As they are the only active compounds identified in this study, this indicates that conformation **III** is likely to reflect the favourite geometry adapted by compounds **A** and **B** in the active site of the enzyme, where it is expected that the compounds are present as only one conformer. However, it is striking that compounds **3b**/**3d** and **B** present such a difference in activity (**3b**: 36% @ 1 μM, **3d**: 25% @ 1 μM vs **B**: 70% @ 1 μM). It should be noticed that the corresponding methoxy derivatives **3a** and **3c** are inactive, while methoxy compound **A** has a similar biological activity as the hydroxy compound **B** (**A**: 63% @ 1 μM). One explanation for this finding it that **A** and **B** might have a different binding mode in the enzyme binding site.

**Figure 4:**
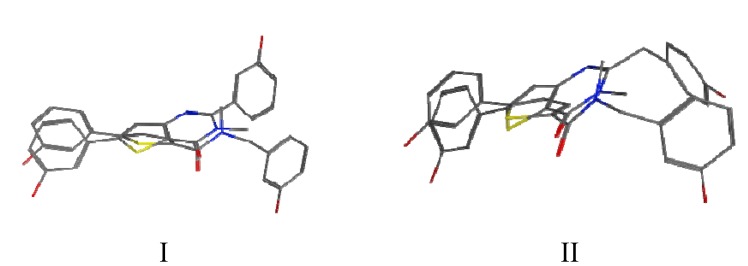
Superimposition between compounds 3b and **B** (Figure 4-I) and between compounds **3d** and **B** (Figure 4-II).

In order to better understand the biological activities, manual superimposition of **3b** and **3d** with **B** were made using MOE 2010.10 (considering the lowest energy for each compound, Figure 4). The thiophene moiety of **3b**/**3d** was superimposed on the one of **B** as well as the corresponding carbonyl and *N*-amide moieties. In the superimposition pictures it can be seen that the compounds do not overlap exactly with each other (however, they show a better hit than the other conformers). The hydroxyphenyl in the 5 position of the fused thiophenes **3b**/**3d** superimposes well on **B**. The main differences concern the second hydroxyphenyl/benzyl which is in a completely different position and the presence of the nitrogen N1 of the thienopyrimidinone (absent in **A**/**B**). It might be that this polar N with hydrogen bond acceptor activity is located in a lipophilic pocket or that there is no space in this area to accept the closed thienopyrimidinone ring. These differences might explain the contrasting biological activities observed for **3b**/**3d** and **B**.

**Figure 5:**
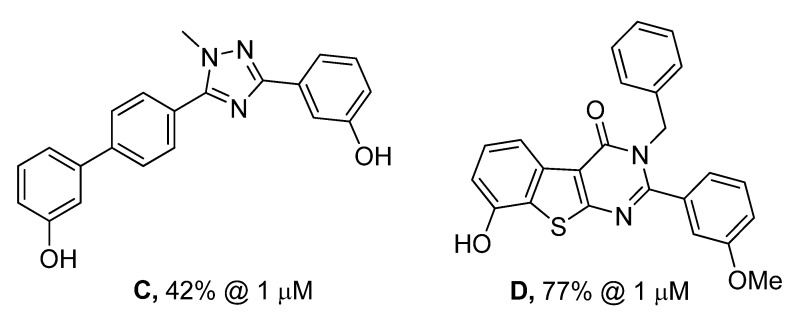
Described analogues of **3b**/**3d** with rigidified amide bond moiety.

In Figure 4, it can also be seen that the planarity induced by the rigidified compounds is comparable to the planarity of the amide and therefore does not seem to have an impact on the potency of the compounds. In this series of compounds the planar triazole derivative **C** (Figure 5), a cyclised bioisostere of the amide function, was synthesized and was also a moderate 17β-HSD2 inhibitor with inhibitory activity in the same range as **3b**/**3d** (C: 42% @ 1 μM, [31]).

In order to investigate the role of the anisole moiety linked to the thienopyrimidinone, compounds **5a**,**c**, without OMe-Ph and with OMe-Ph on the O next to the thiophene, respectively, both derived from rigidification of conformer **I** or **III**, were synthesized. Derivative **5a**, without OMe-Ph was inactive, indicating the importance of this group, which certainly in case of **A** is able to interact with the active site. Compound **5c**, with the OMe-Ph at the O next to the thiophene, was also inactive. This anisole moiety, which covers a different area in the active site, might induce a steric clash, preventing the binding of the compound in the enzyme binding site. It might indicate that the binding cavity is elongated and cannot accommodate globular compounds. This could also explain the inactivity of compounds **4a**/**4b**, which have a different geometry compared to **3b**/**3d**. 

The thienopyrimidinones **3b**/**3d** show a structural similarity to compound **D** [41], previously described as 17β-HSD1 inhibitor (17β-HSD1: 95% inh @ 1 μM, 17β-HSD2: 77% inh @ 1 μM). **D** differs from **3b**/**3d** in the geometry of the pyrimidinone and the OH-Ph fused to the thiophene. As observed for **D**, **3b**/**3d** also shows a slightly better 17β-HSD1 inhibition profile. 

The synthesis of the different rigidified conformers highlights a difference in the reactivity of the amino group depending on its position on the phenyl ring or on the thiophene moiety. All experiments show that the amino group on the thiophene is more reactive than the amino group at the phenyl in spite of the fact that phenyl and thiophene are considered to have similar chemical properties.

## 3. Experimental

Chemical names follow IUPAC nomenclature. Starting materials were purchased from Aldrich, Acros, Combi-Blocks or Fluka and were used without purification. Flash column chromatography (FC) was performed on silica gel (70–200 *μ*m), and reaction progress was monitored by TLC on Alugram SIL G/UV254 (Macherey-Nagel). Visualization was accomplished with UV light.

^1^H-NMR and ^13^C-NMR spectra were measured on a Bruker AM500 spectrometer (at 500 MHz and 125 MHz, respectively) at 300 K in acetone-*d*_6_ or DMSO-*d*_6_. Chemical shifts are reported in *δ* (parts per million: ppm), by reference to the hydrogenated residues of deuteriated solvent as internal standard: 2.05 ppm (^1^H-NMR) and 29.8 and 206.3 ppm (^13^C-NMR) for acetone-*d*_6_, 2.50 ppm (^1^H-NMR) and 39.5 ppm (^13^C-NMR) for DMSO-*d*_6_ and 7.26 ppm (^1^H-NMR) and 77.2 ppm (^13^C-NMR) for CDCl_3_. Signals are described as br (broad), s (singlet), d (doublet), t (triplet), dd (doublet of doublets), ddd (doublet of doublet of doublets), dt (doublet of triplets) and m (multiplet). All coupling constants (*J*) are given in Hertz (Hz). Melting points (mp) were measured in open capillaries on a Stuart Scientific SMP3 apparatus and are uncorrected.

Mass spectrometry was performed on a TSQ® Quantum (ThermoFisher, Dreieich, Germany). The triple quadrupole mass spectrometer was equipped with an electrospray interface (ESI). The purity of the compounds was assessed by LC/MS. The Surveyor®-LC-system consisted of a pump, an auto sampler, and a PDA detector. The system was operated by the standard software Xcalibur®. A RP C18 NUCLEODUR® 100-5 (3 mm) column (Macherey-Nagel GmbH, Dühren, Germany) was used as stationary phase. All solvents were HPLC grade. In a gradient run the percentage of acetonitrile (containing 0.1% trifluoroacetic acid) was increased from an initial concentration of 0% at 0 min to 100% at 15 min and kept at 100% for 5 min. The injection volume was 15 µL and flow rate was set to 800 µL/min. MS analysis was carried out at a needle voltage of 3,000 V and a capillary temperature of 350 °C. Mass spectra were acquired in positive mode from 100 to 1,000 *m/z* and UV spectra were recorded at the wave length of 254 nm and in some cases at 360 nm. All tested compounds exhibited ≥95% chemical purity. Compounds **6** [20], **8** [22], **11a**–**b** [20] were prepared according to previously described procedures.

### 3.1. General Procedure for Suzuki — Miyaura Coupling (***2a**, **17***) — Method A

A solution of **2a** or **17** (1 eq.), 3-methoxyphenyl boronic acid (1.5 eq.), cesium carbonate (3 eq.) and tetrakis(triphenylphosphine)palladium(0) (0.02 eq.) in oxygen free DME/EtOH/H_2_O solution (3 mL, 1:1:1) was heated under microwave irradiation at 150 °C/150 W for 15 min. After cooling to room temperature, the mixture water/EtOAc (2 mL, 1:1) was added to quench the reaction. The solution was extracted three times by EtOAc (3 × 10 mL). The organic layer was dried over MgSO_4_, filtered and the solution was concentrated under reduced pressure. The residue was purified by silica gel column chromatography using *n*-hexane and EtOAc as eluent.

### 3.2. General Procedure for Cleavage of Ethers ***1b**–**4b*** — Method B

To a solution of **1a**–**4a** (1 eq.) in CH_2_Cl_2_ (10 mL) was added dropwise boron fluoride-dimethyl sulfide complex BF_3_.SMe_2_ (3 eq./each methoxy group) at 0 °C. After 1 h at 0 °C, the ice bath was removed and the reaction mixture was stirred overnight at room temperature. The solution was quenched by addition of 10 mL MeOH. After 30 min, the solvent were concentrated under reduced pressure at 35 °C and the residue was triturated with cold water. The aqueous layer was extracted three times with EtOAc (3 × 15 mL), the organic layer was dried over MgSO_4_, filtered and the solution concentrated under reduced pressure. The residue was purified by silica gel column chromatography using *n*-hexane and EtOAc as eluent or triturated in a mixture of diethylether/petroleum ether and filtered off.

*Methyl 3-Amino-5-(3-methoxyphenyl)thiophene-2-Carboxylate* (**6**). To a solution of 3-chloro-3-(3-methoxyphenyl)prop-2-enenitrile (3.56 g, 18.40 mmol) and dry potassium carbonate (3.05 g, 22.08 mmol) in dry NMP (20 mL) was added dropwise under N_2_ atmosphere methyl thioglycolate (2.01 mL, 22.08 mmol). The reaction mixture was heated at 60 °C overnight. After cooling, the reaction mixture was poured into ice water solution and stirred. The aqueous layer was extracted three times with EtOAc (3 × 30 mL). The organic layer was washed once with water, dried over MgSO_4_, filtered and the solution was concentrated under reduced pressure. The residue was purified by silica gel column chromatography (*n*-hexane/EtOAc 70:30) to afford 6 as a yellow solid. Yield: 50%. Mp: 72–74 °C. ^1^H-NMR (CDCl_3_) δ 3.84 (s, 3H), 3.85 (s, 3H), 5.47 (br, 2H), 6.76 (s, 1H), 6.90 (dd, *J* = 1.9, 8.2 Hz, 1H), 7.10 (t, *J* = 1.9 Hz, 1H), 7.16–7.19 (m, 1H), 7.30 (t, *J* = 8.0 Hz, 1H);^ 13^C-NMR (CDCl_3_) δ 51.5, 55.6, 111.8, 114.8, 115.9, 118.8, 130.2, 134.9, 149.2, 154.4, 160.2, 165.2. LC-MS (ESI): [M+H]^+^ = 264.29.

*Methyl 3-{[(1E)-(dimethylamino)methylidene]amino}-5-(3-methoxyphenyl)thiophene-2-carboxylate* (**7**)*.* To a solution of methyl 3-amino-5-(3-methoxyphenyl)thiophene-2-carboxylate **6** (250 mg, 0.95 mmol) in EtOH (10 mL) was added *N*-dimethoxymethyl-*N*,*N*-dimethylamine (189 µL, 1.42 mmol). The reaction mixture was heated under microwave irradiation at 100 °C/80 W for 30 min. The solution was concentrated under reduced pressure to give **7** as an orange oil. Yield: 99%. The title compound was used for the next step without further purification.

*3-(3-Methoxybenzyl)-6-(3-methoxyphenyl)*thieno[3,2-*d*]pyrimidin*-4(3H)-one* (**1a**). A solution of **7** (302 mg, 0.95 mmol) and 3-methoxybenzylamine (146 µL, 1.14 mmol) in DMF (4 mL) was heated under microwave irradiation at 100 °C/80 W for 30 min. Water was added to the solution to quench the reaction. The aqueous layer was extracted three times with EtOAc (3 × 15 mL). The organic layer was washed once with brine and once with water, dried over MgSO_4_, filtered and the solution was concentrated under reduced pressure. The residue was purified by silica gel column chromatography using *n*-hexane and EtOAc as eluent (*n*-hexane/EtOAc 60:40) to afford **1a** as a pale yellow solid. Yield: 31%. Mp: 144–146 °C. ^1^H-NMR (CDCl_3_) δ 3.78 (s, 3H), 3.90 (s, 3H), 5.29 (s, 2H), 6.88 (dd, *J* = 2.3, 8.2 Hz, 1H), 6.99–7.05 (m, 3H), 7.28 (t, *J* = 8.0 Hz, 1H), 7.36–7.44 (m, 3H), 7.68 (s, 1H), 8.48 (s, 1H); ^13^C-NMR (CDCl_3_) δ 49.6, 55.4, 55.8, 112.5, 114.1, 114.7, 116.2, 119.5, 120.9, 122.1, 123.2, 130.7, 131.3, 135.3, 139.4, 150.1, 152.6, 157.4, 158.9, 161.0, 161.4. LC-MS (ESI): [M+H]^+^ = 379.31.

*3-(3-Hydroxybenzyl)-6-(3-hydroxyphenyl)*thieno[3,2-*d*]pyrimidin*-4(3H)-one* (**1b**). Synthesized according to Method B using **1a** (52 mg, 0.14 mmol) and BF_3_.SMe_2_ (88 µL, 0.84 mmol). The residue was triturated in a mixture of diethyl ether/petroleum ether to afford **1b** as a pale brown solid. Yield: 82%. Mp: 270–272 °C. ^1^H-NMR (DMSO-*d*_6_) δ 5.15 (s, 2H), 6.67 (ddd, *J* = 0.7, 2.4, 8.0 Hz, 1H), 6.71 (t, *J* = 1.7 Hz, 1H), 6.77 (d, *J* = 7.7 Hz, 1H), 6.87 (dt, *J* = 2.0, 7.2 Hz, 1H), 7.14 (t, *J* = 7.8 Hz, 1H), 7.17–7.19 (m, 1H), 7.27–7.32 (m, 2H), 7.76 (s, 1H), 8.60 (s, 1H), 9.44 (s, 1H), 9.78 (s, 1H); ^13^C-NMR (DMSO-*d*_6_) δ 48.4, 112.8, 114.2, 114.7, 116.8, 117.0, 118.0, 120.9, 121.4, 129.7, 130.5, 133.6, 138.1, 149.8, 151.5, 156.2, 157.6, 157.7, 158.0. LC-MS (ESI): [M]^+^ = 350.84.

*8-Bromo-4H-3,1-benzoxazin-4-one* (**9**). A solution of 2-amino-3-bromobenzoic acid (250 mg, 1.16 mmol) and triethyl orthoformate (2 mL) was heated under microwave irradiation at 160 °C/80 W for 15 min. After cooling, the precipitate formed was collected by filtration and washed with petroleum ether to afford **9** as colorless needles. Yield: 61%. Mp: 314–316 °C. ^1^H-NMR (acetone-*d*_6_) δ 7.57 (t, *J* = 7.9 Hz, 1H), 8.14 (s, 1H), 8.17 (dd, *J* = 1.5, 7.9 Hz, 1H), 8.20 (dd, *J* = 1.5, 8.0 Hz, 1H);^ 13^C-NMR (acetone-*d*_6_) δ 121.7, 122.2, 128.6, 130.8, 140.9, 144.6, 152.1, 158.4.

*8-Bromo-3-(3-methoxybenzyl)quinazolin-4(3H)-one* (**10**). A solution of **9** (100 mg, 0.44 mmol) and 3-methoxybenzylamine (73 mg, 0.53 mmol) in 2 mL dry toluene was heated at reflux overnight. After cooling, the solvent was removed under reduced pressure. The residue was triturated with diethyl ether and filtered off to give **10** as a colorless solid. Yield: 66%. Mp: 139–140 °C. ^1^H-NMR (acetone-*d*_6_) δ 3.78 (s, 3H), 5.27 (s, 2H), 6.88 (ddd, *J* = 0.6, 2.6, 8.4 Hz, 1H), 6.99–7.02 (m, 1H), 7.04 (t, *J* = 2.1 Hz, 1H), 7.28 (t, *J* = 8.0 Hz, 1H), 7.45 (t, *J* = 7.9 Hz, 1H), 8.11 (dd, *J* = 1.5 Hz, 7.8 Hz, 1H), 8.23 (dd, *J* = 1.5 Hz, 8.0 Hz, 1H), 8.53 (s, 1H); ^13^C-NMR (acetone-*d*_6_) δ 50.1, 55.5, 114.2, 114.7, 120.9, 123.1, 124.8, 127.1, 128.7, 130.7, 138.7, 139.1, 146.9, 149.1, 160.8, 161.0. LC-MS (ESI): [M]^+^ = 345.46, 347.40.

*3-(3-Methoxybenzyl)-8-(3-methoxyphenyl)quinazolin-4(3H)-one* (**2a**). Synthesized according to Method A using **10** (100 mg, 0.29 mmol) and 3-methoxyphenylboronic acid (66 mg, 0.43 mmol). The residue was purified by silica gel column chromatography (*n*-hexane/EtOAc 70:30) to afford **2a** as colorless oil. Yield: 98%. ^1^H-NMR (acetone-*d*_6_) δ 3.75 (s, 3H), 3.81 (s, 3H), 5.23 (s, 2H), 6.85 (ddd, *J* = 0.8, 2.6, 8.3 Hz, 1H), 6.94 (ddd, *J* = 1.0, 2.7, 8.3 Hz, 1H), 6.98–7.01 (m, 1H), 7.04 (t, *J* = 2.1 Hz, 1H), 7.16 (ddd, *J* = 1.0, 1.5, 7.6 Hz, 1H), 7.21 (dd, *J* = 1.6, 2.5 Hz, 1H), 7.25 (t, *J* = 7.9 Hz, 1H), 7.33 (t, *J* = 8.0 Hz, 1H), 7.56 (t, *J* = 7.8 Hz, 1H), 7.81 (dd, *J* = 1.6, 7.4 Hz, 1H), 8.27 (dd, *J* = 1.6, 8.0 Hz, 1H), 8.36 (s, 1H); ^13^C-NMR (acetone-*d*_6_) δ 49.9, 55.56, 55.58, 113.5, 114.1, 114.8, 117.4, 120.9, 123.7, 123.9, 126.8, 127.7, 129.6, 130.7, 135.8, 139.4, 140.3, 140.9, 146.4, 147.5, 160.1, 161.0, 161.5. LC-MS (ESI): [M]^+^ = 372.77.

*3-(3-Hydroxybenzyl)-8-(3-hydroxyphenyl)quinazolin-4(3H)-one* (**2b**). Synthesized according to Method B using **2a** (125 mg, 0.31 mmol) and BF_3_.SMe_2_ (196 µL, 1.86 mmol). The residue was triturated in diethyl ether to afford **2b** as a colorless solid. Yield: 97%. Mp: 155–158 °C. ^1^H-NMR (acetone-*d*_6_) δ 5.43 (s, 2H), 6.84 (ddd, *J* = 0.8, 2.4, 8.1 Hz, 1H), 6.99–7.04 (m, 5H), 7.20–7.23 (m, 1H), 7.36–7.40 (m, 1H), 7.90 (t, *J* = 7.7 Hz, 1H), 8.01 (dd, *J* = 1.5, 7.5 Hz, 1H), 8.40 (dd, *J* = 1.5, 8.1 Hz, 1H), 8.43 (br, 1H), 8.67 (br, 1H), 9.48 (s, 1H);^ 13^C-NMR (acetone-*d*_6_) δ 52.6, 116.5, 117.2, 117.4, 120.7, 121.6, 121.8, 127.9, 130.5, 130.9, 131.6, 135.2, 136.3, 136.5, 136.7, 137.9, 152.1, 158.7, 158.9, 159.3. LC-MS (ESI): [M]^+^ = 344.81.

*3-Amino-5-(3-methoxyphenyl)thiophene-2-carboxamide* (**11a**). A solution of sodium sulfide nonahydrate Na_2_S.9H_2_O (24 g, 100 mmol) in DMF (100 mL) was heated at 40 °C for 30 min. To this solution 3-chloro-3-(3-methoxyphenyl)prop-2-enenitrile (9.68 g, 50 mmol) in DMF (20 mL) was added. After 2 hours at 50 °C, 2-chloroacetamide (8.42 g, 100 mmol) in DMF (20 mL) was added to the reaction mixture. The solution was stirred overnight at 50 °C. Freshly prepared EtONa in EtOH (2.30 g of sodium in 30 mL of absolute EtOH) was added to the reaction mixture, which was stirred and kept at 50 °C for 2 h. After cooling to room temperature, the mixture was poured into ice water solution under stirring. The aqueous layer was extracted three times with EtOAc (3 × 50 mL). The organic layer was washed once with water, dried over MgSO_4_, filtered and the solution was concentrated under reduced pressure. The residue was purified by silica gel column chromatography (EtOAc 100%) to afford **11a** as a pale brown solid. Yield: 21%. Mp: 104–106 °C. ^1^H-NMR (acetone-*d*_6_) δ 3.85 (s, 3H), 6.28 (br, 2H), 6.67 (br, 2H), 6.93 (s, 1H), 6.95 (ddd, *J* = 0.9, 2.6, 8.3 Hz, 1H), 7.16 (t, *J* = 2.1 Hz, 1H), 7.22 (ddd, *J* = 0.9, 1.6, 7.7 Hz, 1H), 7.34 (t, *J* = 8.1 Hz, 1H);^ 13^C-NMR (acetone-*d*_6_) δ 60.0, 111.1, 116.3, 119.37, 119.43, 123.2, 135.3, 140.4, 154.2, 157.0, 165.4, 166.2. LC-MS (ESI): [M]^+^ = 248.94.

*2,6-Bis(3-methoxyphenyl)*thieno[3,2-*d*]pyrimidin*-4(3H)-one* (**12**). A solution of 3-amino-5-(3-methoxyphenyl)thiophene-2-carboxamide **11a** (200 mg, 0.80 mmol), 4-methoxybenzaldehyde (117 µL, 0.96 mmol) and concentrated hydrochloric acid 37% (250 µL) was refluxed for 2 hours. After cooling, the precipitate was collected by filtration, rinsed with cold MeOH and washed twice with diethyl ether to give **12** as a pale brown solid. Yield: 61%. Mp: 241–243 °C. ^1^H-NMR (DMSO-*d*_6_) δ 3.86 (s, 3H), 3.88 (s, 3H), 7.03 (ddd, *J* = 1.3, 2.5, 7.9 Hz, 1H), 7.14 (ddd, *J* = 0.9, 2.7, 8.3 Hz, 1H), 7.36 (t, *J* = 1.9 Hz, 1H), 7.37–7.47 (m, 3H), 7.71 (t, *J* = 1.8 Hz, 1H), 7.74 (ddd, *J* = 0.9, 1.5, 7.7 Hz, 1H), 7.81 (s, 1H);^ 13^C-NMR (DMSO-*d*_6_) δ 56.0, 56.1, 112.3, 113.5, 116.0, 118.2, 119.2, 120.7, 121.1, 122.0, 130.2, 131.0, 134.4, 134.5, 151.5, 155.1, 158.5, 158.7, 160.1, 160.6. LC-MS (ESI): [M+H]^+^ = 365.26.

*2,6-Bis(3-methoxyphenyl)-3-*methylthieno[3,2-*d*]pyrimidin*-4(3H)-one* (**3a**). Methyl iodide (20 µL, 0.32 mmol) was added to a solution of **12** (75 mg, 0.21 mmol) and potassium carbonate (58 mg, 0.42 mmol) in dry CH_3_CN (2 mL). The reaction mixture was refluxed for 3 h. After cooling to room temperature, the reaction was stopped by adding water. The aqueous layer was extracted three times with EtOAc (3 × 5 mL). The organic layer was dried over MgSO_4_, filtered and the solution was concentrated under reduced pressure. The residue was purified by silica TLC using *n*-hexane and EtOAc as eluent (*n*-hexane/EtOAc 60:40) to afford **3a** as a colorless solid. Yield: 48%. Mp: 148–150 °C. ^1^H-NMR (CDCl_3_) δ 3.54 (s, 3H), 3.871 (s, 3H), 3.873 (s, 3H), 6.96 (ddd, *J* = 0.8, 2.5, 8.2 Hz, 1H), 7.05–7.08 (m, 2H), 7.11 (dt, *J* = 1.2, 7.8 Hz, 1H), 7.23 (t, *J* = 2.0 Hz, 1H), 7.30 (ddd, *J* = 1.0, 1.4, 7.7 Hz, 1H), 7.37 (t, *J* = 8.0 Hz, 1H), 7.44 (t, *J* = 7.6 Hz, 1H), 7.51 (s, 1H).^ 13^C-NMR (CDCl_3_) δ 34.2, 55.4, 55.5, 112.1, 113.5, 114.9, 115.9, 119.0, 120.2, 120.8, 121.2, 130.1, 130.3, 134.5, 136.3, 152.5, 156.5, 157.8, 158.5, 159.9, 160.1. LC-MS (ESI): [M+H]^+^ = 379.29.

*2,6-Bis(3-hydroxyphenyl)-3-*methylthieno[3,2-*d*]pyrimidin-4(3H*)-one* (**3b**). Synthesized according to Method B using **3a** (27 mg, 0.08 mmol) and BF_3_.SMe_2_ (51 µL, 0.48 mmol). The residue was triturated in a mixture of diethyl ether/petroleum ether to afford **3b** as a pale pink solid. Yield: 57%. Mp: 303–304 °C. ^1^H-NMR (DMSO-*d*_6_) δ 3.38 (s, 3H), 6.87 (dt, *J* = 2.0, 7.2 Hz, 1H), 6.94 (ddd, *J* = 0.8, 2.5, 8.1 Hz, 1H), 7.00 (t, *J* = 1.7 Hz, 1H), 7.04–7.06 (m, 1H), 7.19–7.20 (m, 1H), 7.27–7.36 (m, 3H), 7.74 (s, 1H), 9.78 (br, 2H); ^13^C-NMR (DMSO-*d*_6_) δ 33.8, 112.7, 115.1, 116.79, 116.81, 117.0, 118.7, 119.8, 120.8, 129.6, 130.5, 133.7, 136.2, 151.2, 156.4, 157.3, 157.6, 157.99, 158.02. LC-MS (ESI): [M]^+^ = 350.85.

*2-(4-Methoxybenzyl)-6-(3-methoxyphenyl)thieno*[3,2-*d*]pyrimidin-*4(3H)-one* (**13**). To a solution of 3-amino-5-(3-methoxyphenyl)thiophene-2-carboxamide **11a** (100 mg, 0.40 mmol) in dry THF (3 mL) was added Et_3_N (67 µL, 0.48 mmol) followed by 4-methoxyphenylacetyl chloride (73 µL, 0.48 mmol). The reaction mixture was stirred at room temperature for 1 h. The solvent was removed under reduced pressure before NaOH 2N (1.5 mL) and DMF (1.5 mL) were added to the residue. The solution was refluxed for 30 min, cooled to room temperature and poured into cold water. The precipitate formed was filtered off, washed with water and diethyl ether to give **13** as a colorless solid. Yield: 49%. Mp: 258–261 °C. ^1^H-NMR (DMSO-*d*_6_) δ 3.71 (s, 3H), 3.83 (s, 3H), 3.89 (s, 2H), 6.87 (d, *J* = 8.6 Hz, 2H), 7.00–7.03 (m, 1H), 7.28 (d, *J* = 8.6 Hz, 2H), 7.33–7.42 (m, 2H), 7.79 (s, 1H), 12.59 (br, 1H);^ 13^C-NMR (DMSO-*d*_6_) δ 50.0, 60.3, 60.6, 114.6, 116.5, 118.3, 118.6, 119.2, 120.6, 123.7, 125.0, 133.6, 135.2, 135.8, 139.1, 155.8, 163.4, 163.9, 165.0. LC-MS (ESI): [M+H]^+^ = 379.22.

*2-(4-Methoxybenzyl)-6-(3-methoxyphenyl)-3-*methylthieno[3,2-*d*]pyrimidin-4(3H)-one (**3c**). Methyl iodide (11 µL, 0.18 mmol) was added to a solution of **13** (46 mg, 0.12 mmol) and potassium carbonate (66 mg, 0.24 mmol) in dry CH3CN (2 mL). The reaction mixture was refluxed for 3 hours. After cooling to room temperature, the reaction was stopped by adding water. The aqueous layer was extracted three times with EtOAc (3 × 5 mL). The organic layer was dried over MgSO_4_, filtered and the solution was concentrated under reduced pressure. The residue was purified by preparative TLC using *n*-hexane and EtOAc as eluent (*n*-hexane/EtOAc 60:40) to afford **3c** as a pale yellow solid. Yield: 74%. Mp: 133–134 °C. ^1^H-NMR (CDCl_3_) δ 3.61 (s, 3H), 3.87 (s, 3H), 3.96 (s, 3H), 4.27 (s, 2H), 6.95 (d, *J* = 8.7 Hz, 2H), 7.02–7.05 (m, 1H), 7.25 (d, *J* = 8.7 Hz, 2H), 7.30–7.32 (m, 1H), 7.34–7.40 (m, 1H), 7.45 (t, *J* = 7.7 Hz, 1H), 7.57 (s, 1H);^ 13^C-NMR (CDCl_3_) δ 30.9, 42.0, 55.5, 55.6, 112.3, 114.7, 115.2, 119.2, 120.8, 121.1, 126.8, 129.6, 130.5, 134.8, 152.5, 156.7, 157.9, 158.7, 159.1, 160.3. LC-MS (ESI): [M+H]^+^ = 393.28.

*2-(4-Hydroxybenzyl)-6-(3-hydroxyphenyl)-3-*methylthieno[3,2-*d*]pyrimid*in-4(3H)-one* (**3d**). Synthesized according to Method B using **3c** (35 mg, 0.01 mmol) and BF_3_.SMe_2_ (56 µL, 0.54 mmol). The residue was triturated in a mixture diethyl ether/petroleum ether to afford **3d** as a pale brown solid. Yield: 98%. Mp: 308–310 °C. ^1^H-NMR (DMSO-*d*_6_) δ 3.46 (s, 3H), 4.16 (s, 2H), 6.72 (d, *J* = 7.1 Hz, 2H), 6.83–6.88 (m, 1H), 7.07 (d, *J* = 7.1 Hz, 2H), 7.17 (br, 1H), 7.26–7.33 (m, 2H), 7.72 (s, 1H), 9.35 (s, 1H), 9.79 (s, 1H);^ 13^C-NMR (DMSO-*d*_6_) δ 30.7, 75.4, 111.8, 113.2, 116.0, 117.2, 117.5, 119.7, 121.3, 125.8, 130.1, 130.9, 134.2, 151.5, 156.7, 157.0, 158.1, 158.5, 159.3. LC-MS (ESI): [M+H]^+^ = 364.89.

*Methyl 2-amino-3-bromobenzoate* (**14**). A solution of 2-amino-3-bromobenzoic acid **8** (1.033 g, 4.78 mmol) and thionyl chloride (30 mL) was heated at reflux for 4 hours. After cooling, the solvent was reduced under reduced pressure. The residue was poured into MeOH at 0 °C. Excess solvent was removed under reduced pressure. The precipitate was filtered and purified by recrystallization in a MeOH/water mixture to afford **14** as a yellow solid. Yield: 89%. Mp: 39–40 °C. ^1^H-NMR (Acetone-*d*_6_) δ 3.87 (s, 3H), 6.57 (t, *J* = 7.8 Hz, 1H), 6.63 (br, 2H), 7.64 (dd, *J* = 1.6, 7.9 Hz, 1H), 7.85 (dd, *J* = 1.5, 8.1 Hz, 1H);^ 13^C-NMR (Acetone-*d*_6_) δ 52.3, 110.8, 112.4, 117.0, 131.6, 138.2, 148.8, 168.5.

*Methyl 3-bromo-2-{[(3-methoxyphenyl)carbonyl]amino}benzoate* (**15**). To a solution of **14** (983 mg, 4.27 mmol) in dry CH_2_Cl_2_ (5 mL) was added Et_3_N (653 µL, 4.70 mmol) followed by 3-methoxyphenylacetyl chloride (600 µL, 4.27 mmol). The reaction mixture was stirred at room temperature overnight. Water was added to the solution to stop the reaction. The aqueous layer was extracted three times with CH_2_Cl_2_ (3 × 20 mL). The organic layer was dried over MgSO_4_, filtered and the solution was concentrated under reduced pressure. The residue was purified by silica gel column chromatography (*n*-hexane/EtOAc 95: 5 to 70:30) to afford **15** as a white viscous oil. Yield: 15%. ^1^H-NMR (acetone-*d*_6_) δ 3.78 (s, 3H), 3.89 (s, 3H), 7.18 (ddd, *J* = 1.0, 2.6, 8.3 Hz, 1H), 7.35 (t, *J* = 7.9 Hz, 1H), 7.46 (t, *J* = 7.7 Hz, 1H), 7.59 (dd, *J* = 1.6, 2.6 Hz, 1H), 7.64 (ddd, *J* = 1.0, 1.5, 7.6 Hz, 1H), 7.90 (dd, *J* = 1.5, 7.8 Hz, 1H), 7.93 (dd, *J* = 1.5, 8.0 Hz, 1H), 9.48 (br, 1H);^ 13^C-NMR (acetone-*d*_6_) δ 52.7, 55.8, 113.7, 118.6, 120.6, 123.8, 128.4, 130.57, 130.59, 131.0, 136.9, 137.2, 137.4, 160.9, 166.1, 166.8.

*8-Bromo-2-(3-methoxyphenyl)quinazolin-4(3H)-one* (**16**). A solution of **15** in formamide (5 mL) was heated at 180 °C for 6 hours. After cooling, the precipitate formed was dispersed with cold water and filtered off. The solid was washed twice with water to give **16** as a pale brown solid. The title compound was used for the next step without further purification. Yield: 81%. Mp: 236–239 °C.^ 1^H-NMR (DMSO-*d*_6_) δ 3.87 (s, 3H), 7.18 (ddd, *J* = 1.0, 2.5, 8.2 Hz, 1H), 7.42 (t, *J* = 7.8 Hz, 1H), 7.49 (t, *J* = 8.0 Hz, 1H), 7.83 (t, *J* = 1.8 Hz, 1H), 7.88 (ddd, *J* = 0.9, 1.4, 7.9 Hz, 1H), 8.14 (dd, *J* = 1.3, 5.4 Hz, 1H), 8.16 (dd, *J* = 1.4, 5.3 Hz, 1H), 12.75 (br, 1H);^ 13^C-NMR (DMSO-*d*_6_) δ 55.4, 112.9, 117.8, 120.3, 122.2, 122.6, 125.7, 127.4, 129.8, 133.6, 137.9, 146.0, 152.6, 159.3, 161.9. LC-MS (ESI): [M]^+^ = 331.54, 333.35.

*2,8-Bis(3-methoxyphenyl)quinazolin-4(3H)-one* (**17**). Synthesized according to Method A using **16** (144 mg, 0.43 mmol) and 3-methoxyphenylboronic acid (98 mg, 0.65 mmol). The residue was purified by silica gel column chromatography (*n*-hexane/EtOAc 70:30) to afford **17** as a colorless solid. Yield: 77%. Mp: 198–201 °C. ^1^H-NMR (DMSO-*d*_6_) δ 3.81 (s, 3H), 3.82 (s, 3H), 6.99 (dd, *J* = 2.0, 8.2 Hz, 1H), 7.09–7.13 (m, 1H), 7.28 (d, *J* = 7.7 Hz, 1H), 7.33 (t, *J* = 2.0 Hz, 1H), 7.39–7.44 (m, 2H), 7.59 (t, *J* = 7.7 Hz, 1H), 7.74–7.77 (m, 2H), 7.90 (dd, *J* = 1.4, 7.4 Hz, 1H), 8.19 (dd, *J* = 1.4, 7.9 Hz, 1H), 12.61 (br, 1H);^ 13^C-NMR (DMSO-*d*_6_) δ 55.0, 55.1, 112.3, 112.9, 116.2, 117.8, 119.8, 121.7, 122.9, 125.4, 126.5, 128.7, 129.7, 134.1, 135.1, 138.4, 139.6, 145.5, 150.8, 158.7, 159.3, 162.4. LC-MS (ESI): [M]^+^ = 358.76.

*2,8-Bis(3-methoxyphenyl)-3-methylquinazolin-4(3H)-one* (**4a**). Methyl iodide (29 µL, 0.47 mmol) was added to a solution of **17** (112 mg, 0.31 mmol) and potassium carbonate (86 mg, 0.62 mmol) in dry CH_3_CN (3 mL). The reaction mixture was refluxed for 3 h. After cooling to room temperature, the solvent was removed under reduced pressure and water was added to the residue to dissolve the inorganic salt. The aqueous layer was extracted three times with EtOAc (3 × 5 mL). The organic layer was dried over MgSO_4_, filtered and the solution was concentrated under reduced pressure. The residue was purified by silica gel column chromatography using *n*-hexane and EtOAc as eluent (*n*-hexane/EtOAc 70:30) to afford **4a** as a colorless solid. Yield: 64%. Mp: 241–244 °C. ^1^H-NMR (acetone-*d*_6_) δ 3.49 (s, 3H), 3.81 (s, 3H), 3.86 (s, 3H), 6.88 (ddd, *J* = 1.0, 2.6, 8.2 Hz, 1H), 7.07 (ddd, *J* = 0.9, 2.6, 8.3 Hz, 1H), 7.23 (dt, *J* = 1.2, 7.7 Hz, 1H), 7.25 (dt, *J* = 1.1, 7.6 Hz, 1H), 7.28–7.29 (m, 1H), 7.30 (t, *J* = 7.8 Hz, 1H), 7.35 (dd, *J* = 1.7, 2.5 Hz, 1H), 7.43 (t, *J* = 8.0 Hz, 1H), 7.59 (t, *J* = 7.7 Hz, 1H), 7.87 (dd, *J* = 1.6, 7.5 Hz, 1H), 8.26 (dd, *J* = 1.6, 7.9 Hz, 1H);^ 13^C-NMR (acetone-*d*_6_) δ 34.4, 55.5, 55.8, 113.8, 114.8, 116.3, 117.3, 121.3, 122.4, 123.5, 126.7, 127.3, 129.5, 130.4, 135.6, 138.3, 139.9, 140.9, 145.5, 156.2, 160.0, 160.6, 163.0. LC-MS (ESI): [M]^+^ = 372.71.

*2,8-Bis(3-hydroxyphenyl)-3-methylquinazolin-4(3H)-one* (**4b**). Synthesized according to Method B using **4a** (65 mg, 0.17 mmol) and BF_3_.SMe_2_ (107 µL, 1.02 mmol). The residue was triturated in diethyl ether to afford **4b** as a colorless solid. Yield: 98%. Mp: 241–244 °C. ^1^H-NMR (acetone-*d*_6_) δ 3.48 (s, 3H), 6.80 (ddd, *J* = 1.0, 2.6, 8.2 Hz, 1H), 6.98 (ddd, *J* = 1.0, 2.4, 8.2 Hz, 1H), 7.11–7.15 (m, 3H), 7.18–7.19 (m, 1H), 7.21 (t, *J* = 7.8 Hz, 1H), 7.33 (t, *J* = 8.1 Hz, 1H), 7.56–7.59 (m, 1H), 7.81 (dd, *J* = 1.7, 7.5 Hz, 1H), 8.25 (dd, *J* = 1.6, 8.0 Hz, 1H), 8.35 (s, 1H), 8.75 (s, 1H); ^13^C-NMR (acetone-*d*_6_) δ 34.4, 115.0, 116.3, 117.6, 118.5, 120.4, 122.3, 122.7, 126.6, 127.3, 129.5, 130.4, 135.6, 138.2, 140.2, 141.0, 145.6, 156.3, 157.7, 158.3, 163.1. LC-MS (ESI): [M]^+^ = 344.77.

### 3.3. General Procedure for the Preparation of Compounds ***18a**–**b*** — Method C

A solution of 3-amino-5-(3-methoxyphenyl)thiophene-2-carboxamide **11a** (4 mmol) or 3-amino-5-(4-methoxyphenyl)thiophene-2-carboxamide **11b** (4 mmol) in formic acid (10 mL) and with catalytic amounts of concentrated sulfuric acid (30 drops) was heated at 50 °C overnight. After cooling, the reaction was quenched with cold water and the solid formed was collected by filtration. The residue was triturated in diethyl ether and filtered off.

*6-(3-Methoxyphenyl)*thieno[3,2-*d*]pyrimidin*-4(3H)-one* (**18a**). Synthesized according to Method C using **11a** (993 mg, 4 mmol) and HCOOH (10 mL). The residue was triturated in diethyl ether to afford **18a** as a pale green solid. Yield: 76%. Mp: 237–245 °C. ^1^H-NMR (DMSO-*d*_6_) δ 3.85 (s, 3H), 7.03 (dt, *J* = 2.4, 6.9, 1H), 7.38–7.43 (m, 3H), 7.87 (s, 1H), 8.17 (s, 1H), 12.55 (br, 1H); ^13^C-NMR (DMSO-*d*_6_) δ 55.4, 111.5, 115.4, 118.5, 121.5, 122.2, 130.5, 133.8, 147.1, 150.4, 157.0, 158.4, 159.8. LC-MS (ESI): [M+H]^+^ = 259.18.

*6-(4-Methoxyphenyl)*thieno[3,2-*d*]pyrimidin-4*(3H)-one* (**18b**). Synthesized according to Method C using **11b** (993 mg, 4 mmol) and HCOOH (10 mL). The residue was triturated in diethyl ether to afford **18b** as a pale green solid. Yield: 98%. Mp: 296–299 °C. ^1^H-NMR (DMSO-*d*_6_) δ 3.82 (s, 3H), 7.04 (d, *J* = 8.9, 2H), 7.69 (s, 1H), 7.78 (d, *J* = 8.9, 2H), 8.14 (s, 1H), 12.45 (br, 1H); ^13^C-NMR (DMSO-*d*_6_) δ 55.4, 114.7, 119.7, 121.2, 125.1, 127.6, 146.9, 150.8, 156.9, 158.6, 160.4. LC-MS (ESI): [M+H]^+^ = 259.19.

### 3.4. General Procedure for the Preparation of Compounds ***19a**–**b*** — Method D

A solution of 6-(3-methoxyphenyl)thieno[3,2-*d*]pyrimidin-4(3*H*)-one (**18a**, 0.58 mmol) or 6-(4-methoxyphenyl)thieno[3,2-*d*]pyrimidin-4(3*H*)-one (**18b**, 0.58 mmol) in phosphorus oxychloride (5 mL) was heated under microwave irradiation at 95 °C/80 W for 20 min. Excess of phosphorus oxychloride was removed under reduced pressure. Ice was added to the residue and the precipitate formed was collected by filtration. The compounds were pure enough and could be used in the next step without further purification.

*4-Chloro-6-(3-methoxyphenyl)*thieno[3,2-*d*]pyrimidine (**19a**). Synthesized according to Method D using **18a** (150 mg, 0.58 mmol) and POCl_3_ (5 mL) afforded **19a** as a brown solid. Yield: 98%. Mp: 153–156 °C. ^1^H-NMR (DMSO-*d*_6_) δ 3.87 (s, 3H), 7.10–7.14 (m, 1H), 7.47 (t, *J* = 8.2, 1H), 7.51–7.54 (m, 2H), 8.28 (s, 1H), 9.03 (s, 1H); ^13^C-NMR (DMSO-*d*_6_) δ 55.5, 111.9, 116.8, 119.2, 120.8, 129.4, 130.7, 132.9, 153.0, 154.3, 154.8, 159.9, 162.5. LC-MS (ESI): [M+H]^+^ = 277.17.

*4-Chloro-6-(4-methoxyphenyl)*thieno[3,2-*d*]*pyrimidine* (**19b**). Synthesized according to Method D using **18b** (150 mg, 0.58 mmol) and POCl_3_ (5 mL) afforded **19b** as a brown solid. Yield: 99%. Mp: 153–156 °C. ^1^H-NMR (DMSO-*d*_6_) δ 3.84 (s, 3H), 7.09 (d, *J* = 8.7, 2H), 7.92 (d, *J* = 8.9, 2H), 8.07 (s, 1H), 8.97 (s, 1H); ^13^C-NMR (DMSO-*d*_6_) δ 55.5, 114.9, 118.7, 124.1, 128.5, 128.8, 152.6, 154.6, 154.7, 161.4, 162.9. LC-MS (ESI): [M+H]^+^ = 277.16.

### 3.5. General Procedure for the Preparation of Compounds **5a**–**b** – Method E

To a solution of 4-chloro-6-(3-methoxyphenyl)thieno[3,2-*d*]pyrimidine **19a** (0.51 mmol) or 4-chloro-6-(4-methoxyphenyl)thieno[3,2-*d*]pyrimidine **19b** (0.51 mmol) in dry DMF (2 mL) was added sodium methanolate in methanol (0.51 mmol) and the reaction mixture was stirred at room temperature for 2 hours. Water was added to quench the reaction. The precipitate formed was collected by filtration and washed once with water and once with petroleum ether.

*4-Methoxy-6-(3-methoxyphenyl)*thieno[3,2-*d*]pyrimidine (**5a**). Synthesized according to Method E using **19a** (140 mg, 0.51 mmol) and NaOMe (0.51 mmol in 2 mL MeOH). The residue was triturated in petroleum ether to afford **5a** as a brown solid. Yield: 91%. Mp: 159–162 °C. ^1^H-NMR (DMSO-*d*_6_) δ 3.86 (s, 3H), 4.13 (s, 3H), 7.07 (dt, *J* = 2.3, 7.4 Hz, 1H), 7.42–7.46 (m, 3H), 8.08 (s, 1H), 8.77 (s, 1H); ^13^C-NMR (DMSO-*d*_6_) δ 54.2, 55.4, 111.8, 115.9, 116.1, 118.8, 120.5, 130.6, 133.6, 151.3, 154.7, 159.9, 162.4, 163.5. LC-MS (ESI): [M+H]^+^ = 273.22.

*4-Methoxy-6-(4-methoxyphenyl)*thieno[3,2-*d*]pyrimidine (**5b**). Synthesized according to Method E using **19b** (140 mg, 0.51 mmol) and NaOMe (0.51 mmol in 2 mL MeOH). The residue was triturated in petroleum ether to afford **5b** as a brown solid. Yield: 40%. Mp: 184–186 °C. ^1^H-NMR (DMSO-*d*_6_) δ 3.83 (s, 3H), 4.12 (s, 3H), 7.08 (d, *J* = 8.8 Hz, 2H), 7.86 (d, *J* = 8.8 Hz, 2H), 7.91 (s, 1H), 8.74 (s, 1H); ^13^C-NMR (DMSO-*d*_6_) δ 54.1, 55.4, 114.8, 115.4, 118.6, 124.8, 128.0, 151.6, 154.7, 160.8, 162.8, 163.3. LC-MS (ESI): [M+H]^+^ = 273.21.

### 3.6. General Procedure for the Preparation of Compounds ***5c**–**d*** – Method F

To a solution of 4-chloro-6-(3-methoxyphenyl)thieno[3,2-*d*]pyrimidine (**19a**, 1 eq.) in dry DMF (2 mL) was added freshly prepared dry sodium 3-methoxyphenolate or sodium 4-methoxyphenolate (1.2 eq.) The reaction mixture was stirred at room temperature for 2 h. Water was added to stop the reaction. Organic layer was washed once with NaOH 2N and once with brine, dried over MgSO_4_, filtered and the solution was concentrated under reduced pressure. The precipitate formed was triturated with a mixture of petroleum ether / diethyl ether and filtered off.

*4-(3-Methoxyphenoxy)-6-(3-*methoxyphenyl)thieno[3,2-*d*]pyrimidine (**5c**). Synthesized according to Method F using **19a** (100 mg, 0.36 mmol) and sodium 3-methoxyphenolate (56 mg, 0.43 mmol). The residue was triturated in petroleum ether to afford 5c as a pale brown solid. Yield: 88%. Mp: 129–132 °C. 1H-NMR (DMSO-*d*_6_) δ 3.78 (s, 3H), 3.87 (s, 3H), 6.92 (dd, *J* = 2.4, 8.2 Hz, 2H), 6.97 (t, *J* = 2.3 Hz, 1H), 7.09 (ddd, *J* = 1.1, 1.3, 8.0 Hz, 1H), 7.39 (t, *J* = 8.2 Hz, 1H), 7.46 (t, *J* = 8.2 Hz, 1H), 7.49–7.51 (m, 2H), 8.16 (s, 1H), 8.70 (s, 1H); ^13^C-NMR (DMSO-*d*_6_) δ 54.4, 55.4, 108.0, 111.86, 111.87, 114.0, 116.1, 116.3, 118.9, 120.5, 130.2, 130.6, 133.4, 152.3, 152.7, 154.6, 159.9, 160.4, 163.1, 163.6. LC-MS (ESI): [M+H]^+^ = 365.26.

*4-(4-Methoxyphenoxy)-6-(3-methoxyphenyl)*thieno[3,2-*d*]pyrimidine (**5d**). Synthesized according to Method F using **19a** (30 mg, 0.11 mmol) and sodium 4-methoxyphenolate (17 mg, 0.13 mmol). The residue was triturated in petroleum ether to afford **5d** as a pale brown solid. Yield: 87%. Mp: 139–146 °C. ^1^H-NMR (DMSO-*d*_6_) δ 3.85 (s, 3H), 3.90 (s, 3H), 6.98–7.01 (m, 3H), 7.15–7.17 (m, 1H), 7.20 (t, *J* = 7.9 Hz, 1H), 7.29 (t, *J* = 2.0 Hz, 1H), 7.36–7.42 (m, 2H), 7.70 (s, 1H), 8.71 (s, 1H); ^13^C-NMR (DMSO-*d*_6_) δ 55.4, 55.6, 112.5, 114.8, 115.4, 117.4, 119.3, 120.0, 122.7, 130.4, 134.3, 145.4, 153.2, 154.8, 157.5, 160.2, 163.6, 164.0. LC-MS (ESI): [M+H]^+^ = 365.25.

## 4. Conclusions

In this study, a series of six thieno[3,2-*d*]pyrimidin-4-ones, four thieno[3,2-*d*]pyrimidines and four quinazolin-4-ones was synthesized and tested for 17β-HSD2 and 17β-HSD1 inhibition. Two compounds **3b** and **3d** were identified as moderate 17β-HSD2 inhibitors, **3b** being the best one with an inhibition of 36% at 1 µM. These compounds resulted from the rigidification of conformer **III**, which can be considered as the active conformer in the enzyme binding site. The inactive globular compounds like **4a**/**4b** or **5c**/**5d** are indicative of the enzyme binding site being rather elongated and are tools to further characterize the 17β-HSD2 active site. Compounds **3b**/**3d** need further optimization focussing on conformer **III** and exchanging the geometry of the pyrimidinone or replacing the polar nitrogen of the pyrimidinone moiety by a carbon or reducing the size of the ring fused to the thiophene. 
